# *TaAbc1*, a Member of Abc1-Like Family Involved in Hypersensitive Response against the Stripe Rust Fungal Pathogen in Wheat

**DOI:** 10.1371/journal.pone.0058969

**Published:** 2013-03-19

**Authors:** Xiaojing Wang, Xiaojie Wang, Yinghui Duan, Shuining Yin, Hongchang Zhang, Li Huang, Zhensheng Kang

**Affiliations:** 1 State Key Laboratory of Crop Stress Biology for Arid Areas and College of Life Sciences, Northwest A&F University, Yangling, Shaanxi, People's Republic of China; 2 Department of Plant Sciences and Plant Pathology, Montana State University, Bozeman, Montana, United States of America; 3 State Key Laboratory of Crop Stress Biology for Arid Areas and College of Plant Protection, Northwest A&F University, Yangling, Shaanxi, People's Republic of China; Nanjing Agricultural University, China

## Abstract

To search for genes involved in wheat (*Triticum aestivum* L.) defense response to the infection of stripe rust pathogen *Puccinia striiformis* f. sp. *tritici* (*Pst*), we identified and cloned a new wheat gene similar to the genes in the Abc1-like gene family. The new gene, designated as *TaAbc1*, encodes a 717-amino acid, 80.35 kD protein. The TaAbc1 protein contains two conserved domains shared by Abc1-like proteins, two trans-membrane domains at the C-terminal, and a 36-amino acid chloroplast targeting presequence at the N-terminal. Characterization of *TaAbc1* expression revealed that gene expression was tissue-specific and could be up-regulated by biotic agents (e.g., stripe rust pathogen) and/or by an abiotic stress like wounding. High-fold induction was associated with the hypersensitive response (HR) triggered only by avirulent stripe rust pathotypes, suggesting that *TaAbc1* is a rust-pathotype specific HR-mediator. Down-regulating *TaAbc1* reduced HR but not the overall resistance level in Suwon11 to CYR23, suggesting *TaAbc1* was involved in HR against stripe rust, but overall host resistance is not HR-dependent.

## Introduction

Plant hypersensitive response (HR), visualized as rapid death of host plant cells at the infection sites, is a means of plant defense response to microbial pathogens [1]–[4]. This process is under intrinsic fine genetic control similar to the programmed cell death (PCD) in animal cells. In mammals, PCD is defined as apoptosis, a complex physiological process that removes unwanted or harmful cells from a body [5]. In plants, HR is triggered by incompatible interactions of host plants and corresponding pathogens. Plant HR may deplete nutrient supply and effectively restrict pathogen growth, especially for biotrophs that only survive and reproduce on living cells. Accompanying rapid cell death, a set of defense responses including cell wall reinforcement, accumulation of reactive oxygen intermediates, antimicrobial compounds and pathogenesis-related proteins is also observed.

Decades of studies in animal and plant systems have revealed that mitochondria play an important role in integrating cell-death signals in regulating PCD and HR [6]. Several genes associated with the death signal pathway through mitochondria have been isolated and characterized. Among those genes, *CABC1*, a human mitochondrial gene that is highly similar to the yeast nuclear gene *Abc1* (for Activity of bc1 complex), has been shown to play an important role in the p53-induced apoptotic pathway [7].

The first *Abc1* gene was cloned and characterized in the yeast *Saccharomyces cerevisiae*, and was shown to suppress the cytochrome b mRNA translation defect [8]. Up to now, *Abc1*-like genes have been identified in nuclei mitochondria and chloroplasts [7]–[9] and have been shown to fulfill diverse functions. The yeast *Abc1* gene was located in the nucleus but has a dual function in mitochondria; it controls the correct folding of cytochrome b and the assembly of bc1 complex in the mitochondrial respiration chain [10]. *Abc1* was lately found to be necessary for yeast coenzyme Q synthesis [11]–[12]. The *Abc1*-like gene family has also been described as a new family of putative protein kinases [13]. A plant *Abc1*-like gene, the *Arabidopsis AtOSA1* gene, located in chloroplasts, was found to be involved in balancing oxidative stress generated by Cd^2+^, hydrogen peroxide (H_2_O_2_), and light [9].

In this study, we were searching for genes involved in wheat (*Triticum aestivum* L.) defense response to the stripe rust pathogen *Puccinia striiformis* f. sp. *tritici* (*Pst*). This fungal pathogen can cause wheat stripe rust disease, and is a worldwide problem threatening wheat production [14]. We isolated a wheat gene that shares the common features of *Abc1*-like genes. Here, we present the results on molecular characteristics of this newly identified wheat *Abc1*-like gene, its expression profiles in response to *Pst* infection and abiotic elicitors and its involvement in wheat defense response to a group of rust fungal pathogens.

## Results

### Identification and isolation of the *TaAbc1* gene

From a cDNA library constructed from the wheat cultivar Suwon11 infected with a virulent *Pst* pathotype CYR31 [15], we selected an EST clone for further characterization in this study due to its strong up-regulated expression in the incompatible interaction of Suwon 11 infected with *Pst* pathotype CYR23. The clone contains a 597-bp cDNA fragment that is highly homologous to the *Abc1* (for activity of bc1 complex) like gene family in rice (*Oryza sativa*) (GenBank accession no. AK120335.1). After database mining, we obtained a 2,675-bp wheat sequence *in silico*. The sequence was highly homologous to a cDNA clone (J013059I22) from *O*. *sativa* Japonica. To verify the gene sequence in wheat, a pair of primers TaAbc1-Fp/Rp (Table 1) was designed and used to amplify the candidate gene from wheat mRNA. A 2,550-bp cDNA sequence was successfully amplified from Suwon11. The sequence includes a 2,154-bp open reading frame (ORF), a 123-bp 5′-untranslated region (UTR), and a 173-bp 3′-UTR predicted using the DNASTAR software (www.dnastar.com). The encoded protein shares an 87% similarity to the Abc1-like protein of *O. sativa* (GenBank accession: BAD22484.1). We designated the wheat gene as *TaAbc1* (GenBank accession: JX944037).

**Table 1:** Primers used in this study.

Primers	Sequence (5′ to 3′)
TaAbc1-Fp	CTCATTTCGCCCCTCTGTTCC
TaAbc1-Rp	GATTTTTACAGGTTTCAACAAGATACAACA
TaEF-F	TGGTGTCATCAAGCCTGGTATGGT
TaEF-R	ACTCATGGTGCATCTCAACGGACT
TaAbc1-fp	GGCAACTGGAGTCGGGTGAT
TaAbc1-rp	TGCGACTATTTGGTTTCCTTGACT
TaAbc1-VIGS-F	ATATTAATTAAGAACTGCTTTCTCATTCATAGGT
TaAbc1-VIGS-R	TATGCGGCCGCCTTAATCATTTGCGGATGAATA

**Figure 1:**
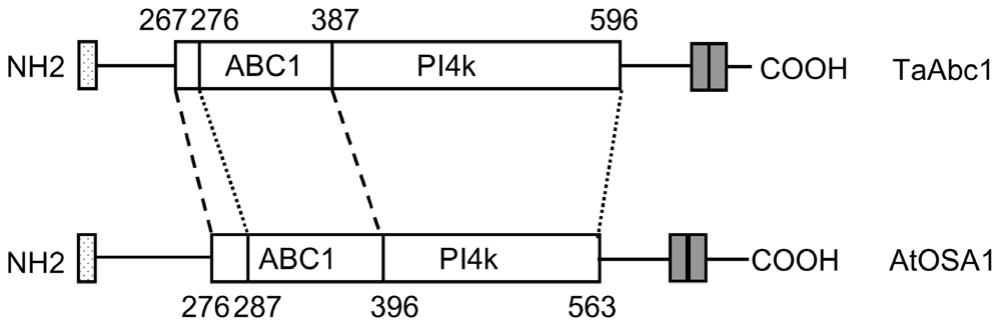
Structure of TaAbc1 protein. With comparison to AtOSA1, an Abc1-like protein in *A. thaliana*, the conserved domains of Abc1 proteins are highlighted. The TaAbc1 protein contained the ABC1 protein family domain (267–387) and putative kinase domain (276–596). The box at N-terminal is the chloroplast targeting presequence. The two filled boxes at C-terminal are two transmembane domains.

The TaAbc1 protein was predicted to have 717 amino acids, a molecular weight of 80.35 kD and an isoelectric point (pI value) of 9.38. The protein includes two conserved domains shared by Abc1-like proteins, two trans-membrane domains located between positions 659–679 and 685–703, and a 36-amino acid N-terminal chloroplast targeting presequence (Fig. 1). The two conserved domains are the domain of 121-amino acids from the region of 267–387 that shares the characteristics of Abc1-like proteins; and the other of 321-amino acids of a putative kinase domain from the region of 276–596.

### *TaAbc1* gene expression

**Figure 2:**
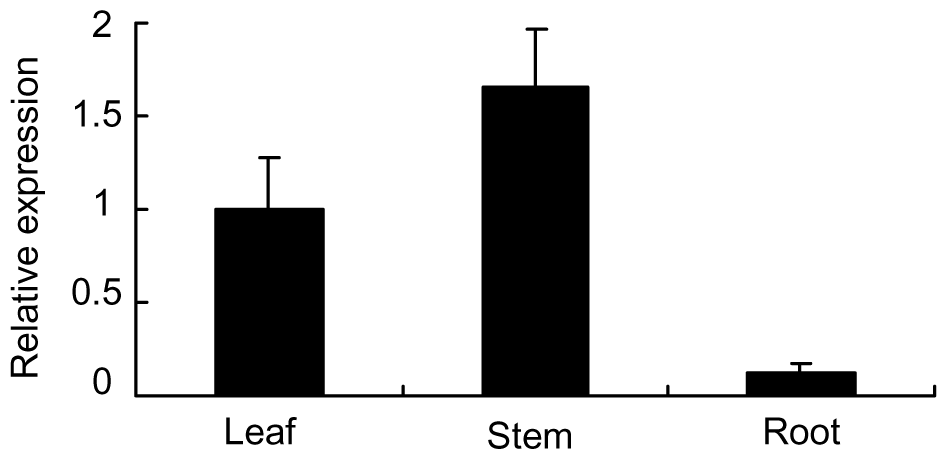
Expression profiles of the *TaAbc1* gene in different wheat tissues. RNAs were isolated from three bulks each of leaf, stem and root tissues that were sampled at the same time from ten 2-week-old plants. Relative expression of *TaAbc1* was normalized to the transcript abundances in leaves (normalized as 1). Error bars represent standard deviation among three biological replicates.

Real-time quantitative PCR was used to measure the relative transcript abundance level of the gene. For assaying the gene expression in different wheat organs, we measured the *TaAbc1* transcripts in leaves, stems and roots at the seedling stage of Suwon11 without pathogen challenge. The gene expression patterns appeared to be tissue-specific. The highest *TaAbc1* transcript abundances were detected in stems. Normalizing the level of *TaAbc1* transcript abundances in leaves as 1, stems had about 50% higher level than that in leaves. In contrast, roots had the lowest -- only about 11% of the level in leaves (Fig. 2).

**Figure 3:**
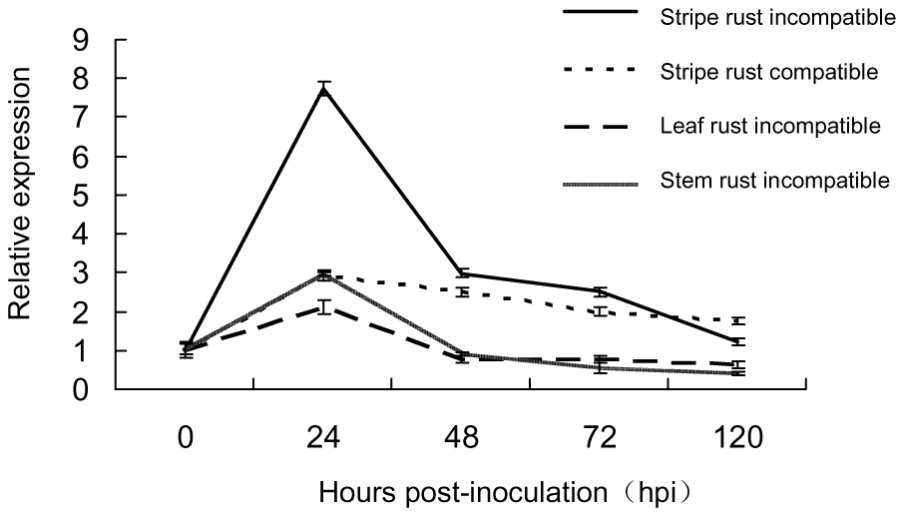
Expression profiles of *TaAbc1* responding to three wheat rust pathogens. Relative *TaAbc1* expressions at five time points of the incompatible (Suwon11/CYR23, Scholar+*Lr47*/PBJL, and Scholar+*Lr47*/QFCSC) and the compatible (Suwon11/CYR31) interactions post rust inoculations. Relative expressions were normalized respectively to the corresponding interaction at 0 hpi (as 1).

To study the expression profiles of *TaAbc1* during the course of rust infections in both incompatible and compatible interactions, we challenged Suwon11 with two *Pst* pathotypes, CYR23 and CYR31. Suwon11 was resistant to CYR23, and susceptible to CYR31, therefore, Suwon11/CYR23 represented an incompatible interaction, and Suwon11/CYR31 a compatible interaction. In addition, we included in the study two additional incompatible interactions with a USA spring wheat genotype Scholar with a leaf rust resistance gene *Lr47*, inoculated with pathotype PBJL of the leaf rust pathogen (*P. triticina*) and pathotype QFCSC of the stem rust pathogen (*P. graminis* f. sp. *tritici*). *TaAbc1* transcript abundances were measured in the leaf tissues collected at 5 time points (Fig. 3) challenged with different rust species and pathotypes in two different wheat cultivars. In both incompatible and compatible interactions, the relative expression of *TaAbc1* was up-regulated and peaked at 24 hours post inoculation (hpi) and declined at 48 hpi. The transcripts remained at higher levels than each of their corresponding 0-hpi-controls at 72 hpi in Suwon11 challenged with both stripe rust pathotypes, while the levels in Scholar+*Lr47* were soon back to the control level at 48 hpi (Fig. 3). The striking difference was that the up-regulated level of *TaAbc1* transcripts was much higher (as much as 7-fold) in the incompatible interaction of Suwon11/CYR23, and increased only up to 2-fold in the rest of the interactions by comparison to each of their corresponding controls at 0 hpi (Fig. 3). By 120 hpi, the *TaAbc1* expression level resumed the level of its 0-hpi-control in the incompatible interaction but was still slightly higher than the 0-hpi-control in the compatible interaction. In summary, *TaAbc1* expression increased and decreased dramatically over the course of 120 hours post inoculation in the incompatible interaction of Suwon11/CYR23; whereas, in the rest of interactions, *TaAbc1* was only slightly increased and decreased over the same time period.

**Figure 4:**
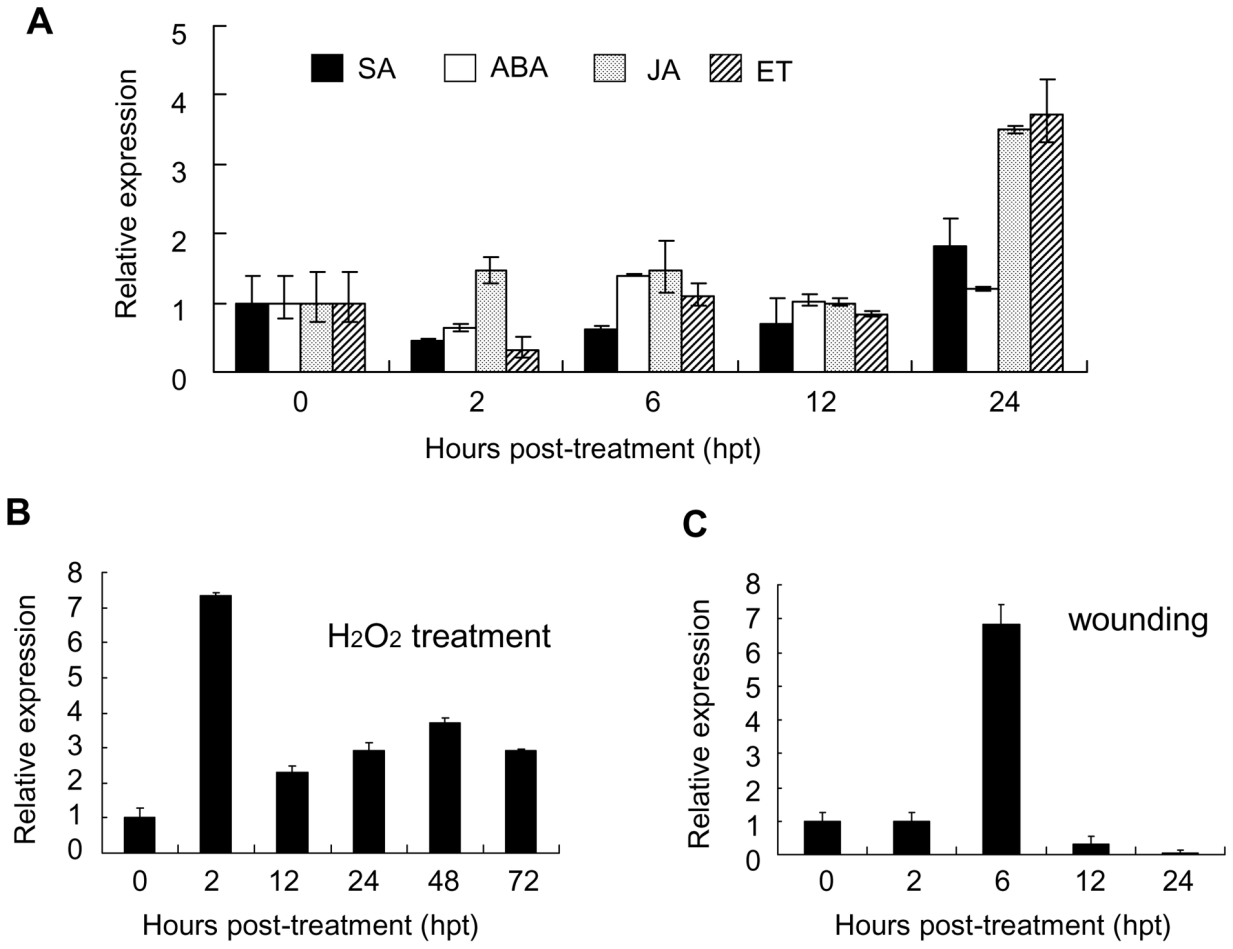
Expression profiles of *TaAbc1* responding to different treatments. A. Response to four exogenous chemicals ET: ethylene, SA: salicylic acid, ABA: abscisic acid, and JA: jasmonate acid, B. Response to 100 mM H_2_O_2_, C. Response to wounding. Relative expressions of each treatment at different time points were normalized to the corresponding treatment at 0 hpt (as 1). Error bars represent the variations among three independent replicates.

Since *TaAbc1* was up-regulated and peaked at 24 hpi with rust challenge in both incompatible and compatible interactions, we asked the question whether the gene can be induced by a signal molecule. We treated Suwon11 with four different signal molecules including salicylic acid (SA), jasmonic acid (JA), ethanol (ET) and abscisic acid (ABA) at the seedling stage. We collected leaf tissues at 5 different time points up to 24 hours post treatment (hpt) and used 0 hpt as the control for each corresponding treatment (Fig. 4A). We normalized the level of *TaAbc1* transcript abundances at 0 hpt as 1 for each treatment. As shown in Fig. 4A, *TaAbc1* was slightly down-regulated at 2 hpt in all except the JA treatment, and then was lingering around the 0 hpt level up to 12 hpt. At 24 hpt, the *TaAbc1* transcript abundances were increased about 3-fold only in the leaves treated with JA and ET, but the magnitudes were significantly smaller than those induced by stripe rust (Fig. 3). No significant changes in *TaAbc1* expression occurred with the SA and ABA treatments.

*TaAbc1* expression was induced by rust inoculations but was unchanged with SA treatment. To search for the signal for *TaAbc1* induction by rust, we treated wheat plants with 100 mM H_2_O_2_ because oxidative burst is one of the earliest host responses upon pathogen detection [16]. Real-time PCR revealed a significant 6-fold increase in *TaAbc1* transcript level in the H_2_O_2_ treated plant as early as at 2 hpt (Fig. 4B), then a decrease to twice the 0-hpt level by 12 hpt. A second small boost of *TaAbc1* level was seen at 48 hpt, about a 3-fold increase in the treated plants than in the control (Fig. 4B). Cell death was visualized at 72 hpt on the H_2_O_2_ treated leaves (data not shown).

To understand the induction by exogenous treatment of JA and ET, we wounded wheat plants by a sterilized scissors and measured *TaAbc1* transcript abundant level at 5 time points as shown in Fig. 4C. As much as a 7-fold increase in *TaAbc1* expression level was detected at 6 hpt, and then the transcripts dramatically declined to an undetectable level by 24 hpt (Fig. 4C).

### Down-regulating *TaAbc1* affects host hypersensitive response triggered by an avirulent pathotype of *Pst*

**Figure 5:**
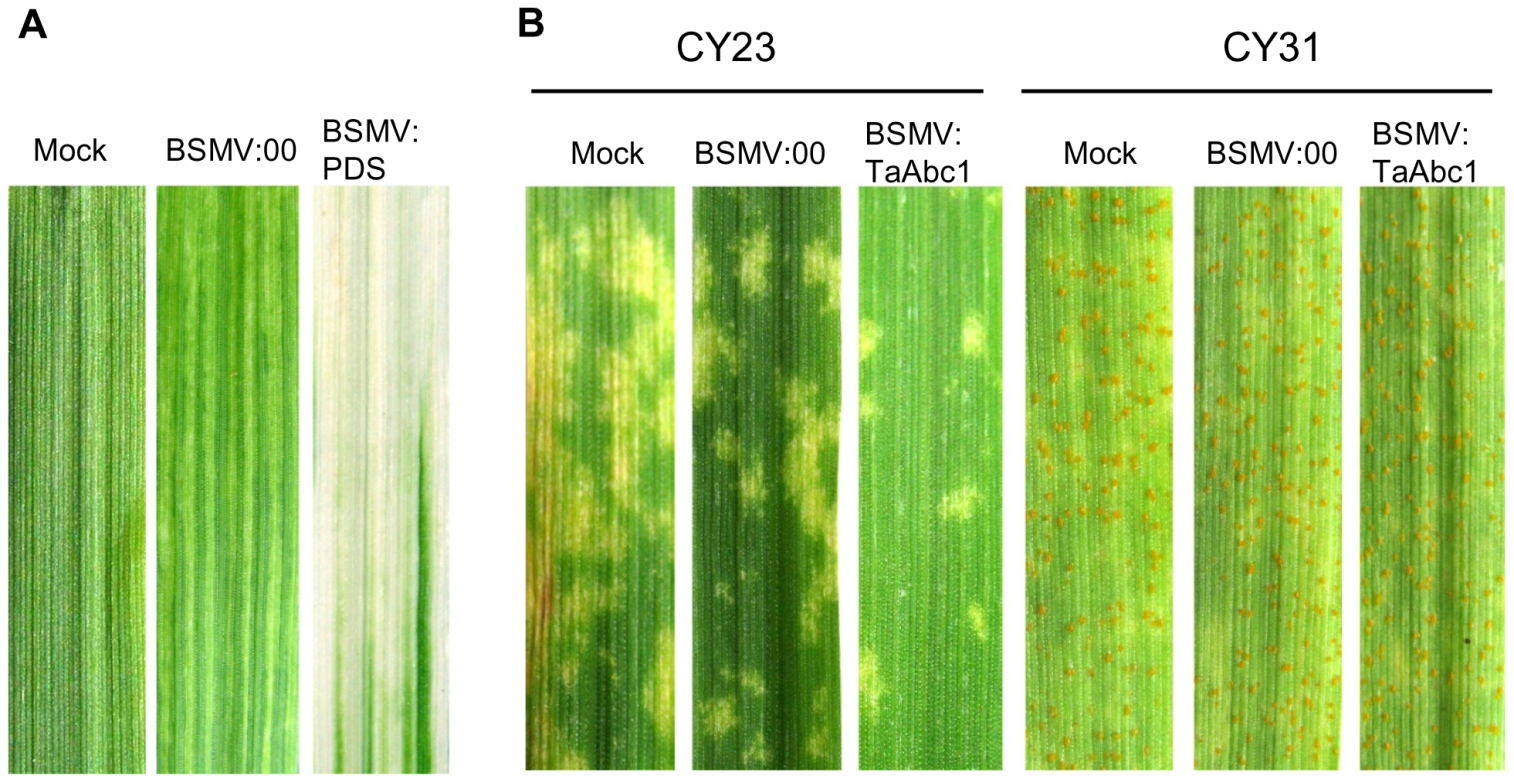
Functional analysis of the *TaAbc1* gene by the BSMV-induced gene silencing assay. 5A: phenotypes of different treatments 9 days post BSMV inoculation. 5B: Stripe rust infection types of Suwon11 15 days post inoculation with CYR23 and CYR31. Mock: wheat leaves inoculated with Fes buffer. BSMV:00: a construct carrying only the BSMV genome; BSMV:PDS: a construct carrying 120-bp wheat phytoene desaturase (*PDS*) gene; BSMV:TaAbc1: a 160-bp fragment of *TaAbc1*.

**Figure 6:**
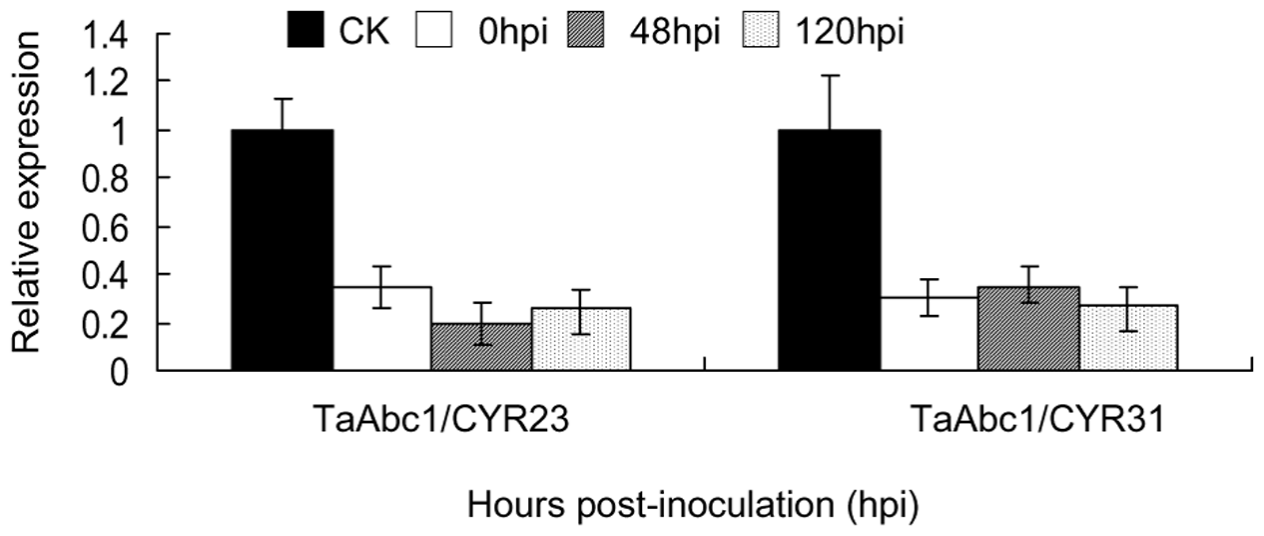
Transcript abundances of *TaAbc1* in Suwon11 during the course of TaAbc1 silencing assay. CK: Suwon11 treated with only BSMV genome. X-axis indicates the stripe rust isolates. Different patterns of bars indicate different time points post rust inoculations. Relative expressions of *TaAbc1* were normalized to the CK at 0 hpi (as 1) in each of the rust inoculations. Error bars represent the variations among three independent replicates.

Rapid induction of *TaAbc1* gene expression was associated with incompatible interaction. To test if *TaAbc1* has any functional role in wheat defense response to stripe rust, we knocked down the endogenous *TaAbc1* gene in Suwon11 using the barley stripe mosaic virus induced gene silencing (BSMV-VIGS) assay. The silencing vector was constructed to contain a 160-bp fragment of *TaAbc1*, designated as BSMV:TaAbc1. As controls, a construct carrying only the BSMV genome, designated as BSMV:00, and a construct carrying a 120-bp wheat phytoene desaturase (*PDS*) gene, designated as BSMV:PDS, were included in the study. In addition, Suwon11 seedlings inoculated with BSMV inoculation buffer were also included as a mock control. Nine days post viral RNA inoculations, plants inoculated with BSMV:PDS started to show photobleaching (Fig. 5A), indicating BSMV induced gene silencing was initiated. Twenty-four each of the *TaAbc1* silenced plants and control plants were then challenged with each of the two stripe rust pathotypes CYR23 and CYR31. Fungal infection types were assessed at 15 days post rust inoculation. As shown in Fig. 5B, Suwon11 had a resistant response to CYR23, exhibited with a high density of necrosis resulting from hypersensitive response (HR) on the mock and BSMV:00 control leaves. *TaAbc1* silenced plants showed the same level of resistance to CYR23 as the controls, but the density of necrosis was significantly less than that on the controls (Fig. 5B). Meanwhile, none of the treatments changed Suwon11 susceptible response to CYR31, and no detectable difference was observed among BSMV:TaAbc1 treated plants and controls. To make sure the silencing assay was effective, leaf tissues were collected from both BSMV:TaAbc1 and BSMV:00 treated plants inoculated with either CYR23 or CYR31 right after fungal inoculation at 0, 48 and 120 hpi. Real-time PCR revealed that *TaAbc1* transcript abundances from all the BSMV:TaAbc1 treated plants were reduced to about 20% to 40% of the relative levels of the BSMV:00 treated control plants at the time of rust inoculation and up to 120 hpi (Fig. 6). The results confirmed the silencing of *TaAbc1* was effective, and knocking down *TaAbc1* reduced HR in Suwon11, triggered by the avirulent pathotype CYR23 (Fig. 5B).

**Figure 7:**
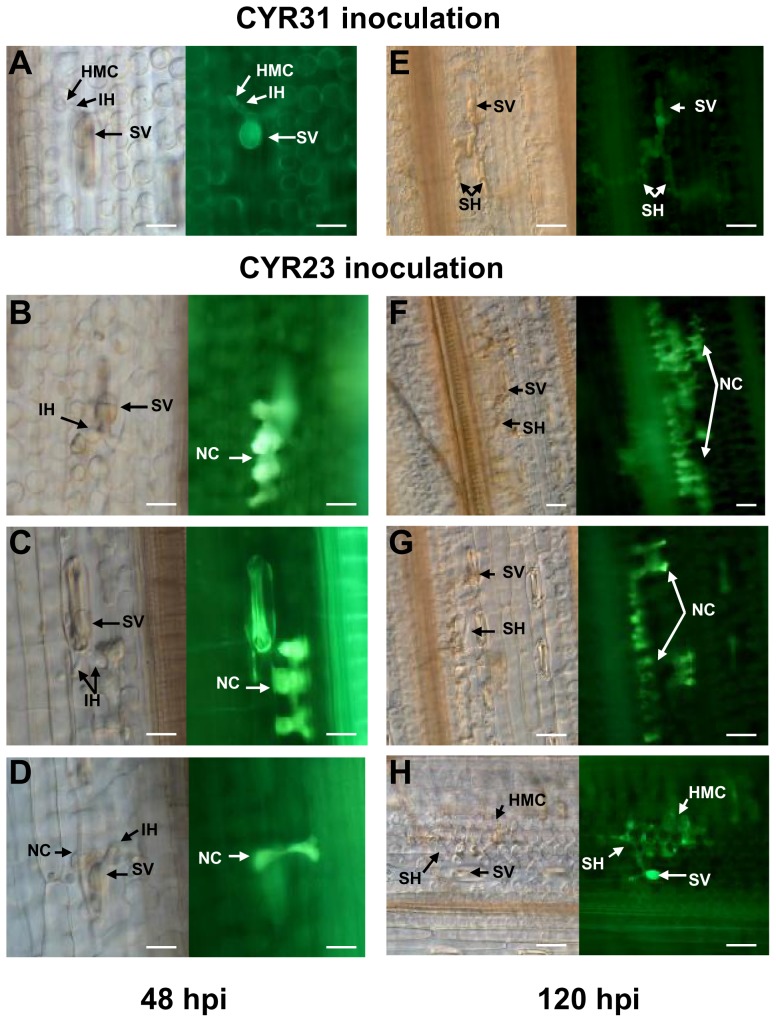
Histological observation of Suwon11 leaves during the course of rust infection. Pictures were taken under an epifluorescence or light microscopy at 48 hpi (left panel) and 120 hpi (right panel). A and E. BSMV:TaAbc1 infected leaves inoculated with CYR31; B and F. mock-inoculated leaves inoculated with CYR23; C and G. BSMV:00 infected leaves inoculated with CYR23; D and H. BSMV:TaAbc1 infected leaves inoculated with CYR23; NC: necrotic cell; SV: substomatal vesicle; IH: initial hyphae; HMC: haustorial mother cell; SH: secondary hyphae; HCWR: host cell wall response. Bars = 50 µm.

Hypersensitive cell death was further examined at 48 and 120 hpi under a florescence microscope as shown in Fig. 7. Auto-florescence was the evidence of cell death, visualized as a necrotic area on leaf surface, and was not detectable on the leaves inoculated with the virulent pathotype CYR31 at either 48 hpi (Fig. 7A) or 120 hpi (Fig. 7E). Strong auto-florescence was seen at the infection sites of CYR23 infection on leaves previously inoculated with only buffer (mock) at 48 hpi (Fig. 7B) and 120 hpi (Fig. 7F), or with BSMV:00 at 48 hpi (Fig. 7C) and 120 hpi (Fig. 7G). Weak auto-florescence was visible at 48 hpi on the leaves in which *TaAbc1* gene was knocked down (Fig. 7D). The florescence areas were also expanded at 120 hpi (Fig. 7H) but were relatively smaller and weaker than those on the leaves of mock and BSMV:00 inoculation (Fig. 7F and 7G). Fungal development and host responses in CYR31-inoculated plants were similar to what have been described for Suwon11–*Pst* compatible interaction [17]. We further measured the size of necrotic area per infection site under a microscope. In an incompatible interaction with CYR23, Suwon11 had an average size of 4 mm^2^ necrotic area per infection site on the control leaves at 48 hpi, and only about 1.6 mm^2^ on the leaves of *TaAbc1* silenced plants, which was significantly (*P*<0.01) smaller than the control (Table 2). At 120 hpi, average sites of necrotic areas became bigger, about 14 mm^2^ per infection site on the control, and 8 mm^2^ on the *TaAbc1* silenced leaves. However, there was no detectable difference observed by the naked eye in resistance level to CYR23 between the controls and *TaAbc1* silenced plants (Fig. 5B). Under microscopy, fungal hyphae were significantly (*P*<0.01) longer in the *TaAbc1* silenced leaves compared with the leaves of the two controls at both 48 and 120 hpi (Table 2). These results suggested that HR had a positive role on restricting fungal infection hyphal growth rates. Knocking down the transcriptional level of *TaAbc1* in Suwon11 reduced the degree of cell death, and in turn, decreased the level of stripe rust resistance during early fungal infection.

**Table 2:** Histological observation and measurement of infection hyphal during the incompatible interaction between Suwon11 and CYR23.

	Hyphal length^y^		Necrotic area per infection site^z^	
Treatment^x^	48 hpi	120 hpi	48 hpi	120 hpi
Control	0.20±0.00a	0.72±0.06a	4.1±0.30a	14.72±6.43a
BSMV:00	0.19±0.00a	0.97±0.02a	4.0±1.4a	13.57±5.73a
BSMV:TaAbc1	0.26±0.02b*	1.76±0.23b*	1.6±0.89b*	8.57±0.15b

x Control, leaves treated with Fes buffer; BSMV:00, leaves treated with BSMV genome with insert; BSMV:TaAbc1, leaves treated with BSMV carrying a fragment of TaAbc1; ^y^mean of values (numbers) ± standard deviation of the hyphal length per colony (unit in 100 µm); ^z^area of dead mesophyll cells around an infection site (unit in 1000 µm^2^, length × width of individual dead spots); a: indicates insignificant difference; b: indicates significant difference at *P*<0. 05; b*: indicates significant difference at *P*<0. 01.

## Discussion

We isolated and characterized a new gene involved in wheat resistance to stripe rust pathogen. The gene resembles the structures of a group of *Abc1*-like genes in animals and plants. In the Arabidopsis genome, there are 17 putative *Abc1*-like genes [9]. Among them, At3g07700 shares 72% amino acid identity with *TaAbc1*. However, so far, only two *Abc1* representatives have been characterized in plants. One is At4g01660, localized in mitochondria [8], [18], and is a homolog of the yeast *Abc1* gene [18]. The encoded protein shares only 28% amino acid identity with TaAbc1.The second one, AtOSA1, shares 46% amino acid identity with TaAbc1 (Fig. S1), and is the first member of *Abc1* family to be localized in the chloroplasts with a function of balancing oxidative stress [9]. Mutants with T-DNA insertion on *AtOSA1* were more sensitive to H_2_O_2_. *TaAbc1* and *AtOSA1* exhibited the same tissue-specific expression patterns that predominantly expressed in leaves and stems, with only a very low level in roots. In addition, both TaAbc1 and AtOSA1 proteins carry an N-terminal chloroplast targeting presequence [9]. These results tend to suggest that the *TaAbc1* may locate and execute its function in chloroplasts.

*TaAbc1* plays an important role in stress response. The gene transcript level followed a complex kinetics, with as much as 2.5∼7-fold induction in response to different stresses, but cell death was only observed on leaves when the induction was as much as 7-fold up-regulated. Our observations imply the existence of a threshold, responding to the combination of stress signals initiated by either a biotic agent (e.g. stripe rust) and/or an abiotic stress like wounding, above which a cell commits itself to suicide. All three rust inoculations which up-regulated the *TaAbc1* transcript abundances in leaves peaked at 24 hpi. However, 7-fold induction was only seen in the incompatible interaction of Suwon11/CYR23. The expression of *TaAbc1* was not affected by SA treatment, suggesting that *TaAbc1* was positioned either up-stream of SA or in an SA-independent manner. Similar levels of 2.5∼3-fold induction were detected in the compatible interaction of Suwon11/CYR31 as well as the other two incompatible interactions of Scholar+*Lr47*/PBJL and Scholar+*Lr47*/QFCSC, where HR was associated with resistance response. It was tempting to speculate that the 2.5∼3-fold induction was an induction level responding to the basal defense triggered by a rust fungal molecule pattern. An exogenous treatment of JA/ET up-regulated *TaAbc1* transcription but the increase was not as much as that detected in the directly wounded plants. JA and ET are two key signaling molecules during plant defenses against necrotrophic pathogens and insects as well as wounding [19]. Induction with exogenous treatment by JA/ET may suggest the involvement of *TaAbc1* in wounding signaling.

Down-regulating *TaAbc1* reduced HR, elicited by the incompatible interaction between Suwon11 and *Pst* pathotype CYR23. In addition, *TaAbc1* transcripts were highly up-regulated by 100 mM H_2_O_2_. These observations invited the hypothesis that *TaAbc1* could be a commonly shared cell-death signal used in wheat defense response to rust fungal pathogens. However, the *TaAbc1* transcript abundance level responding to leaf and stem rust in the two incompatible interactions tested did not support this hypothesis. Our results tend to suggest that *TaAbc1*-mediated HR was rust species and pathotype specific only in the incompatible interactions triggered by stripe rust. It is unclear yet whether all HR seen in incompatible interactions between wheat and stripe rust pathotypes go through *TaAbc1*. With current rather limited observations, we cannot exclude the possibility that *TaAbc1-*mediated HR is *R*-gene specific. Microscopic observations did reveal the timing differences of HR triggered by different rusts. HR associated with resistance to stripe rust fungus appeared at 18 hpi [17], earlier than observed in resistance to leaf or stem rust pathogens in wheat. In general, HR triggered by leaf or stem rust pathogens appeared at 48 hpi (Huang unpublished data). Conserved mechanisms and mediators regulating cell death have been found in animals and plants, but studies also pointed out multiple signaling pathways and different organelles executing programmed cell death [6]. In plants, in addition to mitochondria, the plastid organelles also participate in HR-associated signaling [20], [21]. It is not surprising to learn that HR induced by different rust pathogens used different mediators located in different organelles.

Silencing *TaAbc1* reduced HR triggered by Suwon11/CYR23 incompatible interaction. Significantly longer fungal hyphae were observed under a microscope in *TaAbc1* knocked-down plants compared to the controls. However, there were no visible differences in overall stripe rust resistance between the *TaAbc1* silenced plants and the controls. The results suggest that HR is an important but not an essential defense response to stripe rust in wheat. HR reinforces growth arrest of stripe rust pathogen via depletion of nutrient supply, but overall host defense is not HR-dependent. Accumulating results from studies in wheat and other plant systems show that not all *R*-gene-triggered resistance requires cell death [4]. Potato *Rx*-mediated resistance to potato virus X (PVX) is not associated with HR [22]. Wheat leaf rust resistance gene *Lr34*-mediated resistance has no visible HR [23]. In *Arabidopsis*, the *dnd1* (for defense with no death) mutant expresses resistance when the HR is blocked [24]. Undoubtedly, plant defense response signaling is not a simple straight line; the networks could be parallel, branching or converging. Although HR is a very common rust pathogen resistant phenotype in wheat, it is not an obligatory reaction. Our study suggested that signaling pathways to HR and other defense responses induced by CYR23 in wheat are branched.

The function of *TaAbc1* in resistance toward stripe rust was probably related to reactive oxygen species (ROS). *TaAbc1* transcripts were highly up-regulated by exogenous treatment of 100 mM H_2_O_2_. *AtOSA1* T-DNA-insertion *atosa1* mutants showed increased sensitivity toward H_2_O_2_ and high light. *AtOSA1* transcript levels were up-regulated in wild type after 1 µM cadmium treatment that induced oxidative stress although the mechanism of how the stress is induced by this metal is still obscured [9]. Cd^2+^ can inhibit electron transferring in membranes and induces ROS formation [25]. AtOSA1 probably acts as part of a signal transduction pathway to balance oxidative stress. In the incompatible interaction of Suwon11/CYR23, the generation and accumulation of ROS was detected at infection sites and is associated with the occurrence of HR [17]. In conclusion, we infer that *TaAbc1* plays an important role mediating hypersensitive response. However, the mechanism of *TaAbc1* in wheat defense response and the relationship with ROS need to be further investigated.

## Materials and Methods

### Plant materials

Two wheat (*T*. *aestivum* L.) cultivars Suwon11 (Chinese spring wheat) and Scholar+*Lr47* (USA spring wheat), two stripe rust *Pst* pathotypes CYR23 and CYR31, one leaf rust pathotype PBJL and one stem rust pathotype QFCSC were the biological materials used in this study. Seedlings of Suwon11 and Scholar+*Lr47* were grown and maintained followed the procedure described by Kang and Li [26]. Suwon11, containing the stripe rust-resistance gene *YrSu* [14], is highly resistant to CYR23 but is highly susceptible to CYR31 [27]. Scholar+*Lr47* is highly resistant to leaf rust PBJL and stem rust QFCSC.

### Cloning and sequence analysis

To clone the *TaAbc1* gene, in silico cloning was performed as previously described by Zhang et al. [28]. The rice gene sequence extracted from GenBank was initially used as the seed probe to screen the wheat EST database in GenBank using BLASTN analysis. The sequence was assembled as described by Xia et al [29]. To verify the assembled sequence, primers TaAbc1 (TaAbc1-Fp/Rp, Table 1) were designed to amplify the full length cDNA of *TaAbc1*. The template was a mixture of the first strand cDNA samples extracted from leaves of Suwon11 post inoculated with CYR23 (an incompatible combination) at 12, 24 and 48 hpi. The amplified PCR products were cloned into a pGEM-T Easy Vector (Promega, Madison, WI, USA) and sequenced with an ABI PRISM 3130XL Genetic analyzer (Applied Biosytems, USA).

DNASTAR software (www.dnastar.com), BLAST (http://www.ncbi.nlm.nih.gov/blast/), and ORF Finder (http://www.ncbi.nlm.nih.gov/gorf/gorf.html) were used to analyze the sequence. InterProScan (http://www.ebi.ac.uk/InterProScan/) and PROSITE Scan (http://npsa-pbil.ibcp.fr/cgi-bin/npsa_automat.pl?page=npsa_prosite.html) were used to predict the conserved domains and motifs. The iso-electric point and protein molecular weight were computed using the Compute pI/MW tool (http://www.expasy.org/tools/pi_tool.html). Signal peptide and trans-membrane domains were predicted by TargetIP (http://www.cbs.dtu.dk/services/SignalP/) and TMHMM (http://www.cbs.dtu.dk/services/TMHMM-2.0/). Multiple sequence alignments were created with ClustalW(http://www.ebi.ac.uk/Tools/msa/clustalw2/).

### Rust inoculations and chemical treatments

Stripe rust inoculations were conducted at Northwest A & F University, China. Leaf and stem rust inoculations were conducted at Plant Growth Center, Montana State University, USA. For stripe rust inoculation, freshly collected urediniospores were applied with a paintbrush to the surface of primary leaves of 7-day-old wheat seedlings. After inoculation, plants were incubated for 24 hours in dark in a 100% humidity dew chamber and were subsequently transferred to a growth chamber with a 16 hour photoperiod. Mock stripe rust control inoculation was carried out with ddH_2_O. For leaf and stem rust inoculations, rust urediniospores were suspended in Soltrol 170 isoparaffin (Chempoint, Bellevue, WA). The mocks were carried out with Soltrol 170. For details, visit http://vimeo.com/48605764 to view the videotaped protocols.

For chemical treatments, wheat seedlings were sprayed with 100 µM methyl jasmonate (MeJA), 100 µM ethylene (ET), 100 µM abscisc acid (ABA), 100 µM salicylic acid (SA) and 100 mM hydrogen peroxide (H_2_O_2_), respectively, following Zhang's method [30].

### RNA extraction and quantitative real-time PCR (qRT-PCR)

For tissue-specific expression analyses of *TaAbc1*, intact root, stem, and leaf tissues of 2-week-old wheat seedlings were respectively sampled from the same plants at the same time for the same time point. Sampled tissues for the time-course study were immediately frozen in liquid nitrogen, and stored at −80°C prior to extraction of total RNA. Three independent biological replications were performed for each experiment.

RNA for real-time PCR was isolated with Trizol Reagent (Invitrogen, Carlsbad, CA, USA) following the manufacturer's instructions. First-strand cDNA was synthesized with the GoScript Reverse-Transcription System (Promega Corp., Madison, WI, USA). Primer design and qRT-PCR reactions were conducted as described by Wang [31]. Quantitative real-time PCR was performed using a 7500 Real-Time PCR System (Applied Biosystems, Foster City, CA, USA). The wheat elongation factor *TaEF-1a* gene (GenBank accession number Q03033) was used as an internal control for qRT-PCR analysis. The gene-specific primers, TaEF-F/R (amplifying a 85-bp *TaEF-1a* fragment) and TaAbc1-fp/rp (amplifying a 167-bp *TaAbc1* fragment) for qRT-PCR, are listed in Table 1. Dissociation curves were generated for each reaction to ensure specific amplification. Threshold values (Ct) generated from the ABI PRISM 7500 Software Tool (Applied Biosystems) were used to quantify relative gene expression using the comparative 2^−ΔΔCT^ method [32].

### BSMV-mediated gene silencing

Plasmids used for gene silencing were based on the constructs described by Holzberg and associates [33]. A cDNA fragment (120 bp) of the wheat phytoene desaturase gene *TaPDS* was obtained by reverse-transcriptase polymerase chain reaction (RT-PCR). A RNA-derivative clone (BSMV:PDS) was created using BSMV:GFP (green fluorescent protein) cDNA as the starting material. The *GFP* coding sequence in BSMV: GFP was replaced by a PDS fragment in an antisense orientation resulting in BSMV:PDS. BSMV:TaAbc1 was constructed by the same approach; a 160 bp *TaAbc1* cDNA fragment amplified by a pair of primers TaAbc1-VIGS-F/R(Table 1), derived from the 3′untranslated region was inserted.

Capped in vitro transcripts were prepared from linearized plasmids that contain the tripartite BSMV genome [34] using the mMessage mMachine T7 in vitro transcription kit (Ambion, Austin, TX, USA) following the manufacturer's instructions. The BSMV inoculum was made by combining an equimolar ratio of α, β, and γ transcripts at a 1∶1∶1 ratio with excess inoculation buffer (named as FES) containing a wounding agent. The third leaf of a three-leaf wheat seedling was inoculated with BSMV transcripts by gently rubbing the leaf surface with a gloved finger [33], [35], [36]. As a control, 24 seedlings were inoculated with 1× FES buffer as a mock [37]. Three independent sets of inoculations were performed, with a total of 72 seedlings inoculated for each of the three BSMV viruses (BSMV:00, BSMV:PDS, and BSMV:TaAbc1). Post viral inoculation, wheat plants were maintained in a growth chamber at 23±2°C, and examined for symptoms at regular intervals. Once photobleaching was observed, three independent sets of inoculations including CYR23, CYR31 and sterile water (as a mock), were performed. Infection types of stripe rust were examined at 15 days post rust inoculation. The fourth leaves corresponding to the photobleached areas of BSMV:PDS infected plants were divided for histological observation and real-time PCR assay.

### Histological observation of fungal growth and host response

Wheat leaves infected with BSMV were sampled at 0, 48 and 120 hpi with *Pst* and stained as described by Wang [17]. Cleared leaf segments were examined under an Olympus BX-51 microscope (Olympus Corp., Tokyo) for infection sites and lengths of infection hyphae. Auto-fluorescence of attacked mesophyll cells was observed as a necrotic death area by epifluorescence microscopy (excitation filter, 485 nm; dichromic mirror, 510 nm; and barrier filter, 520 nm). At least 50 infection sites were examined on each of five randomly selected leaf segments per treatment. Only the infection sites with fungal appressoria formation over stomata were considered to have been successfully penetrated. The necrotic leaf area was measured with a calibrated eyepiece micrometer and corresponding areas (square micrometers) calculated according to the formula π × length × width/4. Standard deviations and Tukey's test for statistical analysis were performed with the SPSS software (SPSS, Inc. Chicago, USA).

## Supporting Information

Figure S1 **Amino acid comparison between TaAbc1 and AtOSA1 on the two conserved domains of Abc1-like proteins.**
(TIF)
