# Supplementary figures and images for: TaAbc1, a Member of Abc1-Like Family Involved in Hypersensitive Response against the Stripe Rust Fungal Pathogen in Wheat
Source: PLoS One. 2013 Mar 19;8(3):e58969. doi: 10.1371/journal.pone.0058969 (PMC3602590; doi:10.1371/journal.pone.0058969)

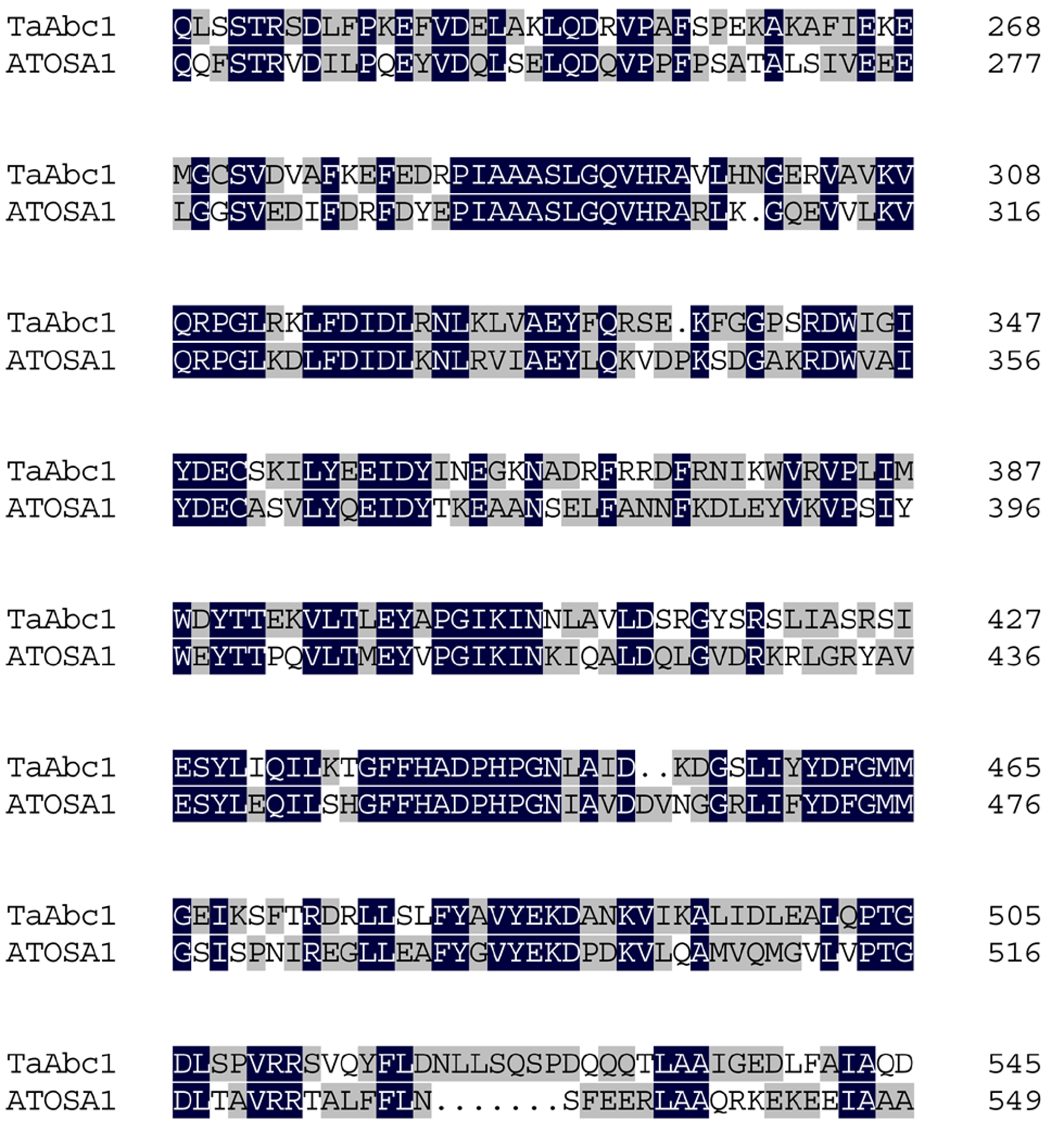

Supplement: Figure S1 — Amino acid comparison between TaAbc1 and AtOSA1 on the two conserved domains of Abc1-like proteins. (TIF) [file pone.0058969.s001.tif]
